# Romosozumab-Induced Epidural Calcification in a Hemodialysis Patient

**DOI:** 10.7759/cureus.95255

**Published:** 2025-10-23

**Authors:** Satoshi Yoshikawa

**Affiliations:** 1 Department of Emergency and Internal Medicine, Rakuwakai Marutamachi Hospital, Kyoto, JPN

**Keywords:** chronic kidney disease, ectopic calcification, hemodialysis, osteoporosis, romosozumab, spine

## Abstract

We report the case of a 75-year-old woman who developed rapidly progressive epidural calcification with vertebral destruction after initiation of romosozumab, followed by regression after the medication was discontinued. The patient, who had a prior history of pyogenic spondylodiscitis and was on maintenance hemodialysis, developed new-onset severe low back pain three months after starting romosozumab. Radiographic evaluation confirmed extensive epidural and perivertebral calcification with vertebral body destruction. A CT-guided drainage provided symptomatic relief, and microbiological cultures were negative for infection. After discontinuation of romosozumab, serial imaging demonstrated progressive regression of calcification, and the patient remained asymptomatic at follow-up without recurrence of pain or evidence of infection.

This case highlights a rare but clinically significant complication of romosozumab therapy, which is ectopic epidural calcification with structural damage in the setting of chronic kidney disease (CKD) and pre-existing spinal pathology. Awareness of this phenomenon is crucial to avoid misdiagnosis as recurrent infection, to prevent unnecessary interventions, and to ensure timely discontinuation of the offending agent. Further recognition may help refine monitoring strategies and guide safer use of osteoanabolic agents in high-risk populations.

## Introduction

Crystal deposition within periarticular and soft tissue structures is a recognized cause of inflammation and pain and is broadly categorized as crystal-induced arthropathy. Among these, hydroxyapatite (HA) crystals, the most common form of basic calcium (Ca) phosphate, may deposit in the articular cartilage, synovium, tendons, intervertebral discs, and vascular walls, leading to inflammation and destructive changes [1].

Romosozumab, a monoclonal antibody against sclerostin, has recently been introduced as an osteoanabolic therapy for osteoporosis [2]. By inhibiting sclerostin, romosozumab enhances bone formation, reduces resorption, and increases bone mineral density [3]. However, concerns remain regarding its potential to promote ectopic calcification, which typically consists of Ca phosphate salts, including hydroxyapatite, particularly among high-risk populations, such as patients with chronic kidney disease (CKD) [3,4]. The U.S. FDA has issued a black box warning due to the increased risk of cardiovascular events [5,6].

Reports of soft-tissue calcification associated with romosozumab are rare. To date, only one case of psoas myositis ossificans after spinal surgery has been described [7]. Here, we describe a hemodialysis patient with a history of spinal infection who developed rapid epidural calcification and destructive vertebral changes following romosozumab therapy that improved after drug discontinuation.

## Case presentation

A 75-year-old woman with a nine-year history of hemodialysis for diabetic nephropathy presented with severe low back pain that impaired her ability to sit independently. She had a history of type 2 diabetes mellitus and had sustained fractures of the right fourth to seventh ribs five years earlier. Four years earlier, she had undergone L2/3-S1 laminoplasty for pyogenic spondylodiscitis with an epidural abscess at the L4/5 level.

Three months before admission, she had initiated monthly subcutaneous injections of romosozumab of 210 mg for osteoporosis, based on a dual-energy X-ray absorptiometry finding showing a femoral neck bone mineral density of 53% of the young adult mean. At initiation, her intact parathyroid hormone (iPTH) level was 107 pg/mL (Table 1).

**Table 1:** Serial laboratory findings of the patient with reference ranges Values are pre-dialysis unless otherwise noted. The samples from day 0, day 21, and two months post-discharge were obtained post-dialysis. Reference ranges are based on institutional standards. Ca: Calcium; Corrected Ca = Measured Ca + 0.8 × (4.0 − albumin (g/dL))

Analyte	One year prior	Three months prior	one month prior	One week prior	At admission (Day 0)	Day 21	Two months post-discharge	Reference range
Corrected Ca (mg/dL)	8.9	8.5	8.5	8.1	8.8	9.4	9.3	8.8–11.0
Inorganic phosphate (IP) (mg/dL)	5.2	7.7	5.1	4.5	2.5	2.0	3.1	2.7–4.5
Magnesium (mg/dL)	-	-	2.5	-	2.1	2.0	-	1.5–2.4
Alkaline phosphatase (ALP) (U/L)	133	82	136	120	-	106	98	38–113
iPTH (pg/mL)	276	107	114	160	-	-	-	10–65
Whole parathyroid hormone (wPTH) (pg/mL)	-	-	-	-	-	-	138	8.3–38.7
White blood cell (WBC) (×10³/μL)	5.2	7.2	12.6	9.5	5.4	5.0	4.2	4.0–8.0
C-reactive protein (CRP) (mg/dL)	0.02	0.06	16.27	0.07	0.05	0.23	0.02	<0.24

**Figure 1:**
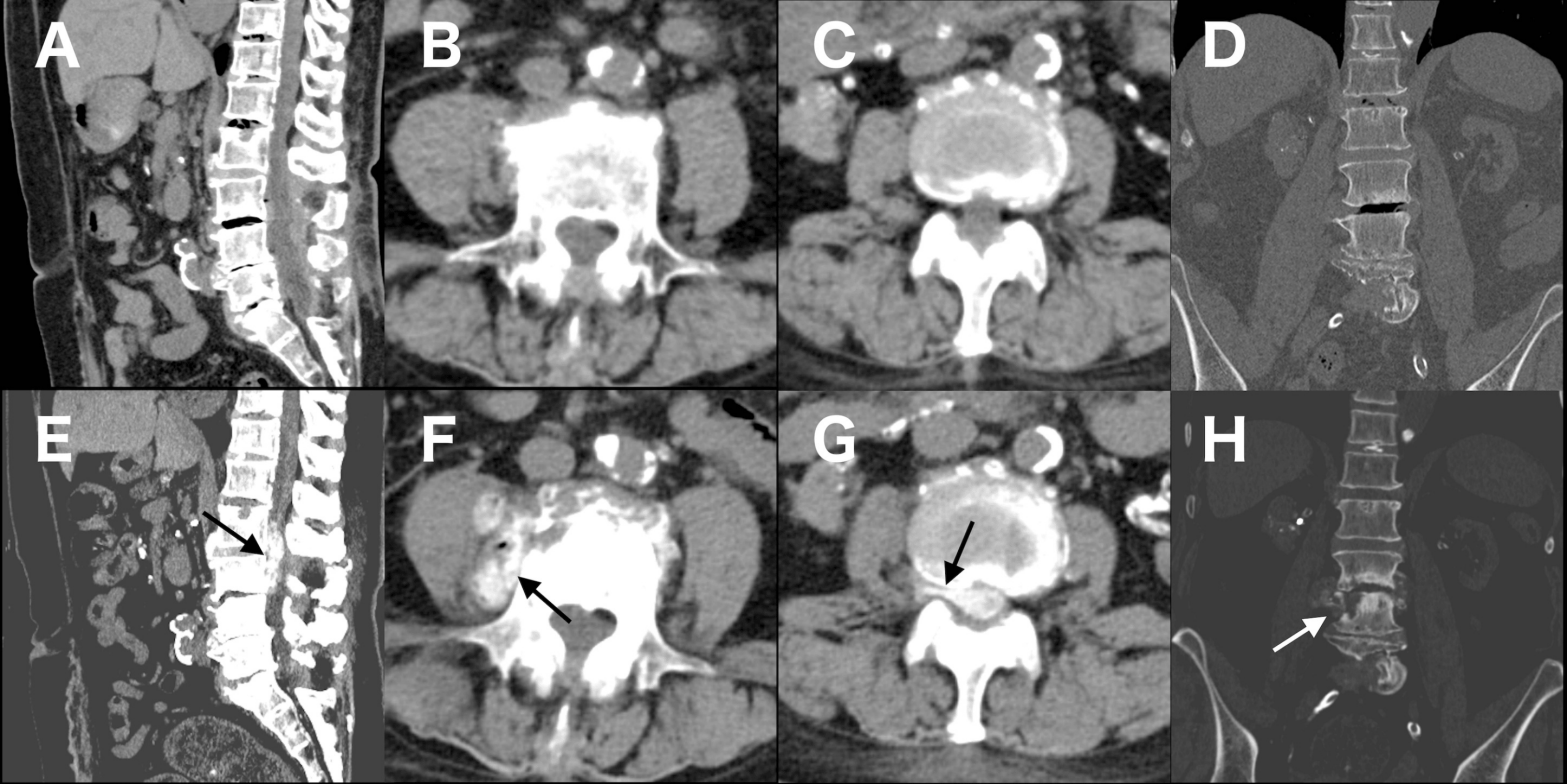
CT findings three months before admission (panels A-D) and on admission (day 0) (panels E-H). A, E: Sagittal images showing epidural calcification at L2–3 (arrow); B, F: Axial images showing perivertebral calcification (arrow); C, G: Axial images showing calcification in the right L2/3 intervertebral foramen (arrow); D, H: Axial images showing destructive changes in the L4 vertebral body (arrow) Panels A, E; B, F; C, G; and D, H represent the same anatomical levels for comparison, demonstrating the progression of calcification and vertebral destruction over time.

One month before admission, the patient was hospitalized for low back pain and right L2 radiculopathy. Although she was afebrile, her C-reactive protein (CRP) level was 16.27 mg/dL (Table 1). The CT revealed a calcified epidural soft tissue lesion at L2-3, arising from the L3/4 intervertebral space, causing severe spinal stenosis. The lesion extended into the right L2/3 intervertebral foramen and the perivertebral region at the L4 level. These findings were not detected on the CT performed three months earlier (Figure 1, panels A-D). The CT-guided needle aspiration of the L3/4 disc space was also performed. Although cultures were negative and polarizing, microscopy revealed neither leukocytes nor crystals; crystal arthropathy was suspected. Her symptoms improved within two weeks with nonsteroidal anti-inflammatory drugs (NSAIDs) alone, without requiring antibiotics.

One day before the present admission, she developed progressive electric-shock-like pain radiating from her lower back to both of her buttocks. Her medications included epalrestat 100 mg, sertraline 50 mg, pitavastatin 2 mg, vonoprazan 10 mg, clopidogrel 75 mg, lemborexant 5 mg, loxoprofen 30 mg, tramadol 50 mg, daily insulin injections, monthly romosozumab 210 mg, saccharated ferric oxide 40 mg, and darbepoetin 10 μg. She was also receiving maxacalcitol 5 μg and upacicalcet 25 μg three times per week.

On admission (day 0), the patient was afebrile and had no overt neurological deficits. Physical examination revealed radiating pain in the lateral aspects of both lower legs upon palpation of the buttocks. Laboratory findings showed a CRP level of 0.05 mg/dL (Table 1). The CT revealed progressive destruction of the L4 vertebral body and enlargement of the calcified epidural soft tissue (Figure 1, panels E-H), compared with the previous study, without fluid in the L3/4 disc space. No progression of calcification was observed in other regions, including the vasculature and coronary arteries. Given the temporal association, drug-related epidural and perivertebral calcification were suspected, and romosozumab was discontinued. The pain was refractory to NSAIDs and acetaminophen.

**Figure 2:**
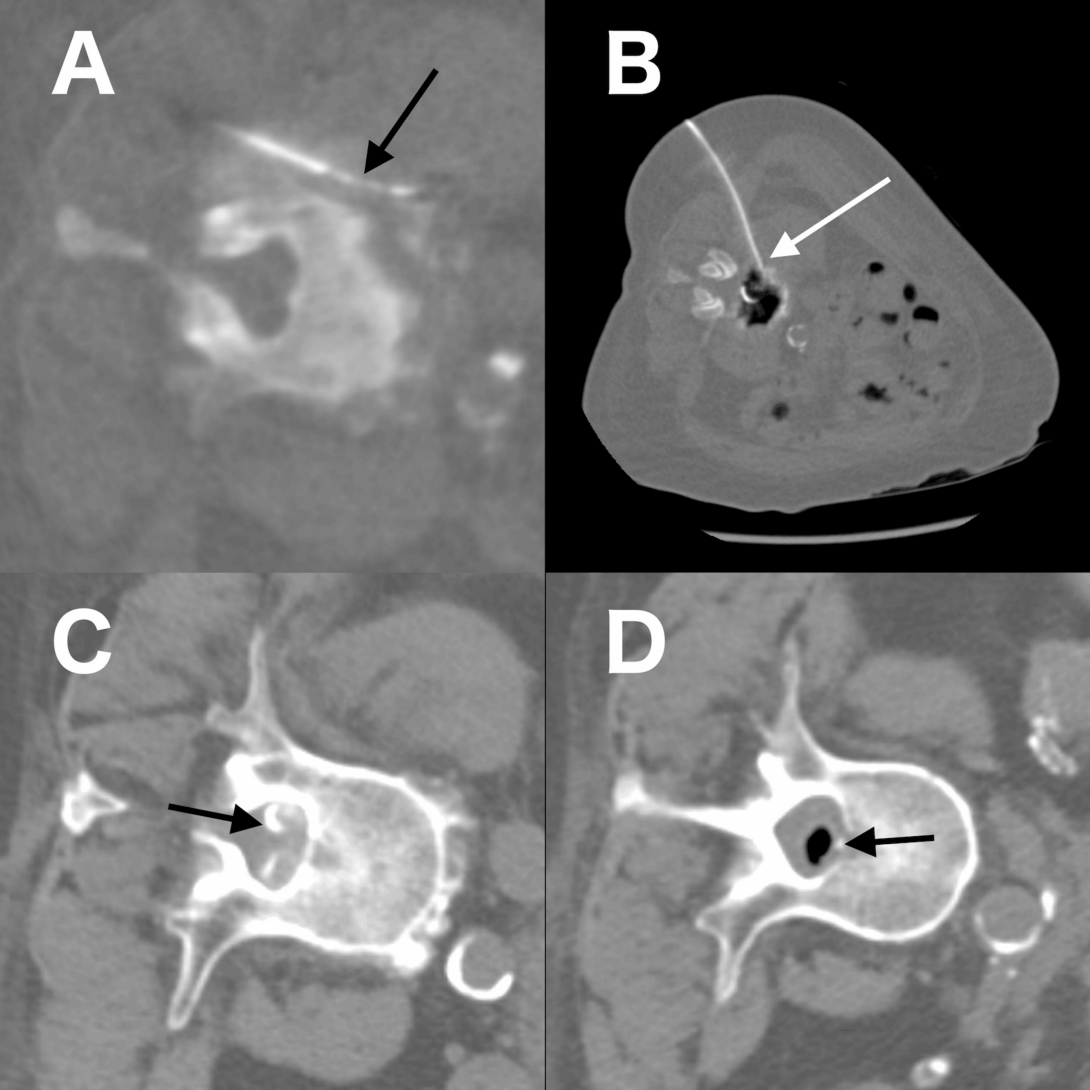
CT-guided intervention findings on day 15 A: Biopsy needle at the L4 perivertebral calcification (black arrow); B: Drainage tube placement (white arrow) in the L3/4 intervertebral disc space; C: Contrast injection through the drainage tube demonstrating partial epidural enhancement at the L3 level (black arrow); D: Epidural air at the L2 level after lavage, indicating epidural decompression and partial communication between the L2/3 disc and epidural spaces (black arrow)

Surgical decompression was considered but deemed unfeasible owing to the patient’s prior laminoplasty. On day 15, a CT-guided biopsy of the L4 perivertebral lesion and drainage tube placement into the L3/4 disc space with lavage were performed. Contrast injection through the drainage tube demonstrated partial epidural enhancement, and epidural air was noted after lavage, indicating epidural decompression and partial communication between the L2/3 disc space and epidural space (Figure 2, panels A-D).

**Figure 3:**
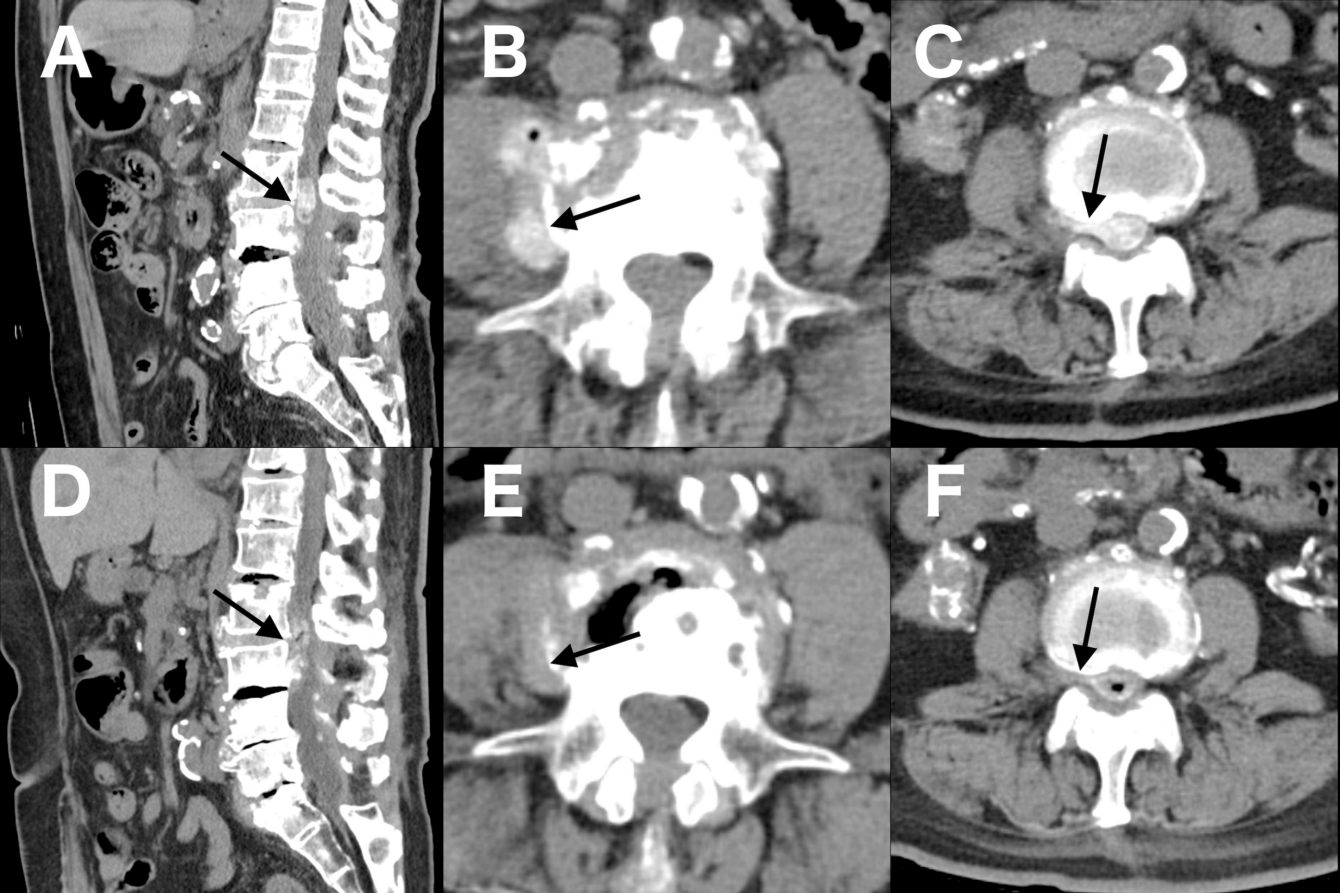
CT Images on day 28 and follow-up CT findings two months after discharge A-C: CT images on day 28 of admission, after removal of drainage tube, showing residual epidural and perivertebral calcifications (black arrows). The perivertebral calcification in panel B was the site of the biopsy performed on day 15. D-F: CT images at the two-month follow-up obtained at the same levels as panels A, B, and C (taken on day 28), respectively, demonstrating the regression of epidural calcification. Notably, the perivertebral calcification visible in panel B disappeared in panel E (black arrows).

Following the procedure, her pain improved, and she regained the ability to sit independently. The patient was discharged on day 43. At the two-month follow-up, she remained asymptomatic, and CT demonstrated further regression of the epidural calcification compared with imaging on day 28 of admission, after removal of the drainage tube (Figure 3, panels A-F). This continued regression supports that the improvement represented an ongoing biological process triggered by discontinuation of romosozumab, rather than a transient mechanical effect of drainage.

**Figure 4:**
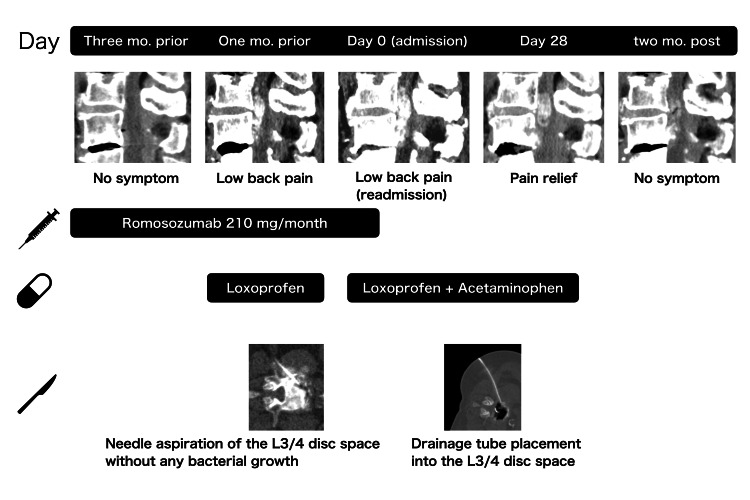
Timeline of romosozumab administration, symptom progression, and radiological changes Serial sagittal CT images show new epidural calcification after initiation of romosozumab, followed by regression after drug discontinuation and CT-guided drainage.

The overall clinical course, including the temporal relationship between romosozumab administration, symptom progression, and radiological changes, is summarized in Figure 4. Serial laboratory testing showed persistently low CRP during admission, stable Ca and inorganic phosphate (IP) levels, and an increase in iPTH from 107 pg/mL at the initiation of romosozumab to 160 pg/mL upon admission (Table 1).

Histopathology revealed von Kossa and Alizarin red positivity in fragmented bone tissue surrounded by osteoblasts. The specimen had been obtained from the perivertebral calcification (Figure 2, panel A), which had almost completely disappeared on follow-up CT (Figure 3, panel E) compared with imaging after drainage tube removal (Figure 3, panel B). Therefore, the fragmented bone tissue was considered most likely related to romosozumab-induced calcification.

## Discussion

This case illustrates the potential of romosozumab to induce rapid ectopic calcification and destructive changes in the spinal epidural space of high-risk patients. Dystrophic and metastatic calcification represent distinct pathophysiological processes. The former occurs in areas of prior tissue injury or necrosis and is typically independent of systemic Ca or phosphate abnormalities, whereas the latter results from ectopic calcification in normal tissues secondary to disturbances in mineral metabolism, such as hypercalcemia and/or hyperphosphatemia, commonly seen in patients with CKD [8].

In this patient, a previous episode of pyogenic spondylodiscitis may have provided a local substrate for dystrophic calcification, while CKD conferred a systemic predisposition to metastatic calcification. However, no epidural or extraskeletal calcification had been observed before romosozumab therapy, and the new calcific lesion appeared exclusively in the epidural and perivertebral space after treatment. These findings indicate that romosozumab acted as a potent trigger that accelerated ectopic calcification in a patient harboring both dystrophic and metastatic susceptibilities.

Romosozumab exerts its effects by neutralizing sclerostin, thereby promoting bone formation and reducing resorption [2,3]. However, sclerostin also plays an inhibitory role in ectopic calcification [3], which is typically composed of Ca phosphate salts, such as HA [4]. In CKD, circulating sclerostin levels are elevated and may function as a compensatory mechanism against ectopic calcification; thus, neutralization of sclerostin may lead to excessive Wnt pathway activation, disrupting bone-mineral homeostasis and predisposing patients to abnormal calcification. Although preclinical studies have not demonstrated ectopic mineralization [3], the rapid development of epidural calcification within three months of therapy and its regression within two months of discontinuation in our patient provide strong evidence of a causal association. Given that serum Ca, phosphate, and PTH levels remained stable during treatment, the regression was unlikely due to correction of systemic mineral imbalance. Rather, the cessation of sclerostin inhibition may have attenuated Wnt pathway activation, allowing partial reversal of the local mineralization process.

Once HA crystals are deposited, they can drive tissue destruction through prostaglandin synthesis, matrix metalloproteinase activation, and cytokine release [1]. Despite progressive destruction, CRP levels remained low, reminiscent of Milwaukee shoulder syndrome, in which HA crystals induce local damage predominantly via non-inflammatory mechanisms [9].

Evidence regarding the use of romosozumab in hemodialysis patients remains limited. A small prospective study [10] reported a slight progression of vascular calcification, supporting concerns in this population. Moreover, pharmacovigilance analyses have described rising PTH levels during treatment [6], which may further affect mineral metabolism.

From a therapeutic perspective, bisphosphonates (BPs) have been reported to inhibit soft-tissue and vascular calcification in both experimental and clinical settings [11]. Among these agents, etidronate, a first-generation BP, exhibits the strongest inhibitory effect on HA formation and has been shown to suppress vascular and coronary calcification in dialysis patients [12]; however, these observations were not derived from randomized controlled trials.

Nitrogen-containing BPs, such as alendronate, have also demonstrated potential anti-calcific effects in vitro and in animal models. However, their affinity for HA is markedly lower than that of etidronate, and their inhibitory effect on crystal formation is consequently weaker [11]. Although clinical studies have yielded inconsistent results, prospective trials of alendronate in patients with CKD have failed to demonstrate a significant reduction in vascular calcification progression [13].

Despite these potential yet limited benefits, BP therapy was not administered in our case because the patient had end-stage renal disease requiring maintenance hemodialysis, in which the use of these drugs is generally contraindicated. In our case, early discontinuation of romosozumab halted disease progression, and CT-guided drainage provided symptom relief. Subsequent regression of calcification paralleled the clinical improvement.

Limitations

This report has several limitations. First, histopathological examination demonstrated von Kossa and Alizarin red positivity in the fragmented bone tissue; however, a definitive pathological classification could not be established. The specimen was obtained from the perivertebral calcified lesion (Figure 2, panel A), which subsequently regressed on follow-up CT (Figure 3, panel E). These findings confirm the presence of ectopic mineralization, but the precise nature of the process remains uncertain. Given the histologic appearance of bone fragments, the deposition of HA, and the clinical background of prior infection and CKD, it is possible that dystrophic calcification, metastatic calcification, and heterotopic ossification each contributed to varying degrees. Acknowledging such uncertainty is essential, as the interplay of local tissue injury, systemic mineral imbalance, and osteogenic signaling after romosozumab exposure may have collectively driven the development of pathological mineralization. Second, polarizing microscopy revealed the absence of crystals in the aspirated fluid. This may be explained by the fact that accurate identification of HA often requires special stains or advanced imaging techniques [14].

## Conclusions

Romosozumab may accelerate ectopic calcification not only in vascular tissues but also in spinal and soft tissue structures, particularly in patients with CKD or a prior spinal injury. This case underscores the importance of recognizing this complication, as it may mimic recurrent infection and lead to unnecessary interventions if not promptly identified. Clinicians should remain vigilant for unexplained epidural calcification or destructive changes in such patients and carefully balance therapeutic benefits against the potential risk of ectopic mineralization. Further recognition of this phenomenon is warranted to refine patient selection and monitoring strategies for high-risk populations. Future research is needed to clarify the mechanisms underlying ectopic calcification during anti-sclerostin therapy and establish preventive approaches that ensure safer use of osteoanabolic agents.
